# Clinical and Immunological Biomarkers for Systemic Lupus Erythematosus

**DOI:** 10.3390/biom11070928

**Published:** 2021-06-22

**Authors:** Haitao Yu, Yasuo Nagafuchi, Keishi Fujio

**Affiliations:** 1 Department of Laboratory Medicine, The First Hospital of Lanzhou University, Lanzhou University, Lanzhou 730000, China; 2 Department of Allergy and Rheumatology, Graduate School of Medicine, The University of Tokyo, Tokyo 113-8655, Japan; FUJIOK-INT@h.u-tokyo.ac.jp; 3 Department of Functional Genomics and Immunological Disease, Graduate School of Medicine, The University of Tokyo, Tokyo 113-8655, Japan

**Keywords:** systemic lupus erythematosus, biomarkers, diagnosis, monitoring, omics

## Abstract

Systemic lupus erythematosus (SLE) is characterized by immune system dysfunction and is clinically heterogeneous, exhibiting renal, dermatological, neuropsychiatric, and cardiovascular symptoms. Clinical and physiological assessment is usually inadequate for diagnosing and assessing pathophysiological processes in SLE. Clinical and immunological biomarkers could play a critical role in improving diagnosis, assessment, and ultimately, control of SLE. This article reviews clinical and immunological biomarkers that could diagnose and monitor disease activity in SLE, with and without organ-specific injury. In addition, novel SLE biomarkers that have been discovered through “omics” research are also reviewed.

## 1. Introduction

Systemic lupus erythematosus (SLE) is a systemic autoimmune disease characterized by aberrant activity of the immune system [1] and presents with a wide range of clinical manifestations, including renal, dermatological, neuropsychiatric, and cardiovascular symptoms [2]. The incidence of SLE is 0.3–31.5 in 100,000 per year, and the adjusted prevalence is approaching, or even exceeding, 50–100 in 100,000 [3]. Unfortunately, there appears to be a trend of increasing SLE prevalence with time [4]. Healthcare-related costs of SLE are related to disease severity and the types of organ(s) involved [5]. Patients with SLE had mean annual costs of about $21,000–$53,000 in the USA [6], and their mean annual direct medical cost was €2600–€4800 in Europe [7].

SLE is an important social and public health problem, as the medication and multidisciplinary approach for treating SLE can only control the symptoms and delay the progression of the disease but cannot cure it completely [1]. It is critical to improve the ability to diagnose SLE early for effective treatment. Therefore, biomarkers, especially immunological biomarkers, have emerged to help better diagnose SLE and assess its pathophysiological processes, with the ultimate goal of improving control of the disease. The aim of the current study was to review immunological biomarkers for SLE diagnosis and pathophysiological process assessment.

## 2. Characteristics of Biomarkers

A biomarker is defined as a measurable indicator of a normal biological process, a pathogenic process, or a response to drug exposure or intervention [8]. Recently, biomarkers were redefined such that they must have a specific physical sign or cellular, biochemical, molecular, or genetic character and can be used to detect and/or monitor a biologic process or morbid state by a qualitative and/or quantitative test [9]. Blood, urine, or tissues can be measured for biomarkers [10]. In recent years, biomarkers have been widely employed in the diagnosis, prediction, assessment, and management of several diseases, such as SLE, diabetes, heart disease, and cancer [11].

**Figure 1:**
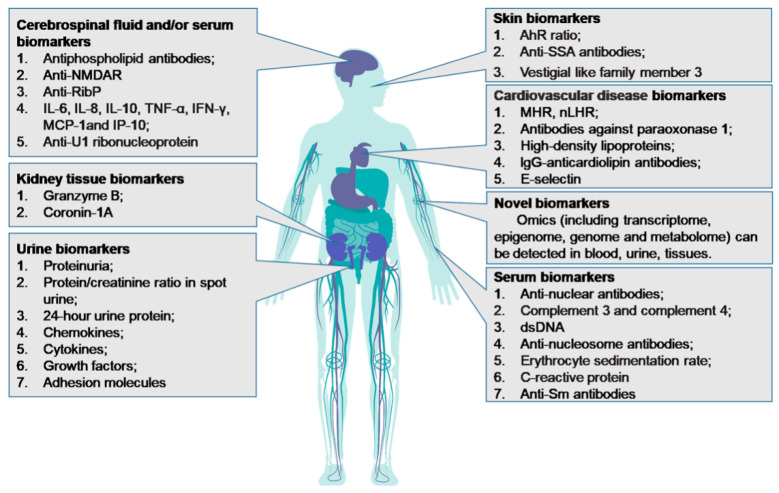
Common biomarkers for SLE and their measurement sites in patients with SLE. AhR ratio: the ratio of aryl hydrocarbon receptor in Th17 cells to that in Treg; anti-NMDAR: antibodies against N-methyl-D-aspartate receptor; anti-RibP: antibodies against ribosomal proteins; anti-SSA: antibodies against Sjogren’s syndrome A; dsDNA: double-stranded DNA; IgG: immunoglobulin G; IFN: interferon; IL: interleukin; IP-10: IFN-γ-inducible protein 10; MCP-1: monocyte chemotactic protein-1; MHR: monocyte-to-high-density lipoprotein cholesterol ratio; nLHR: low-density granulocytes-to-high-density lipoprotein cholesterol ratio; PON1: antibodies against paraoxonase1; Sm: Smith; TNF: tumor necrosis factor.

Biomarkers play a crucial role in diagnosing SLE, classifying SLE complications, assessing SLE disease activity, and reflecting the therapeutic effect of interventions for SLE. Common biomarkers for SLE and their measurement sites in patients with SLE are highlighted in Figure 1. Finding an ideal biomarker for SLE is challenging because it should have the following characteristics: (1) reflect the underlying pathophysiology or treatment target; (2) have reliability, validity, high predictive values, and high sensitivity and specificity; (3) have the ability to monitor SLE activity or flares; (4) are reliably measured in tissues, cells, or fluids, and not be influenced by other factors or comorbidities; and (5) are stable, reproducible, easily detected, and testing should be readily available in most laboratories at a reasonable cost [9,12]. It should be noted that reproducibility and reliability may be affected by laboratory errors, specific techniques, or changes in storage [13]. Because SLE can cause damage to various organs, has a complex pathogenesis, and displays heterogeneous clinical manifestations, one particular biomarker may only reflect one specific aspect of SLE but not be useful for reflecting the state of the disease as a whole [14,15].

## 3. Biomarkers for SLE Diagnosis and Classification

SLE is diagnosed and classified based on a patient’s clinical symptoms, signs, and laboratory biomarkers that reflect immune reactivity and inflammation in various organs. It is necessary to develop consistent classification criteria of SLE for research and clinical diagnosis. The most widely used classification criteria for SLE was established by the American College of Rheumatology (ACR) and contains laboratory biomarkers, including proteinuria, urinary casts, hemolytic anemia with reticulocytosis, white blood cells, lymphocytes, platelets, the presence of Smith (Sm) antibody, antinuclear antibody (ANA), DNA antibody, total complement activity, complement (2, 3, and 4), and lupus erythematosus (LE) cells [16]. The classification criteria were revised by the Diagnostic and Therapeutic Criteria Committee of the ACR in 1997. The revised criteria removed the marker of “LE cell” and added anticardiolipin antibodies as a biomarker for the immunologic criteria [17]. To improve the criteria’s clinical relevance and incorporate new knowledge of the immunological basis of SLE, the Systemic Lupus International Collaborating Clinics (SLICC) group revised and validated the ACR SLE classification criteria in 2012. The SLICC criteria emphasize that at least one immunologic criterion is required for SLE, and ANAs or double-stranded DNA (dsDNA) antibodies can be used for categorizing nephritis compatible with SLE [18]. The SLICC-2012 classification criteria of SLE show improved sensitivity and lower specificity compared to the ACR-1997 classification criteria [18,19]. In 2019, new classification criteria for SLE were developed by the European Alliance of Associations for Rheumatology (EULAR) and the American College of Rheumatology (ACR). Positive ANA is emphasized as an obligatory entry criterion for SLE by the EULAR/ACR-2019 SLE classification, and three immunologic biomarkers (antiphospholipid antibodies, complement proteins, SLE-specific antibodies) and seven clinical indices (constitutional, hematologic, neuropsychiatric, mucocutaneous, serosal, musculoskeletal, renal) are used as additive weighted criteria for SLE [20]. The EULAR/ACR-2019 SLE classification criteria strengthen the role of immunological biomarkers and have a better sensitivity (96.1%) and specificity (93.4%) compared with SLICC-2012 and ACR-1997 [20].

Table 1 categorizes the biomarkers used for SLE classification based on the ACR-1997, SLICC-2012, and EULAR/ACR-2019 criteria. Anti-dsDNA antibodies and hypocomplementemia are required in the classification criteria of SLICC-2012 and EULAR/ACR-2019 but not in the ACR-1997 criteria (Table 1). In patients with early SLE, both EULAR/ACR-2019 and SLICC-2012 criteria are more sensitive than the ACR criteria, and the EULAR/ACR-2019 criteria have excellent specificity [20]. Due to the enhanced sensitivity of the EULAR/ACR-2019, patients with SLE could be classified, diagnosed, and treated early [21,22,23]. Despite the performance of these criteria, SLE diagnosis remains challenging, and some patients with possible SLE disease can still be missed. There are a few reasons for a missed diagnosis of SLE. First, the sensitivity and specificity of current biomarkers are not ideal. Second, high levels of physician skill and experience are required for SLE diagnosis. Finally, few patients with SLE show clinical symptoms in the early stages of disease, making SLE difficult or impossible to diagnose, especially in patients with limited SLE features [24].

## 4. Non-Organ-Specific Biomarkers for SLE

### 4.1. Serum ANA

ANA detected by indirect immunofluorescence (IIF) on HEp-2 cells has long been regarded as a pivotal immunological biomarker in serum for classifying a patient with SLE, as well as assessing eligibility for SLE [25,26,27,28]. The ANA test is included in the ACR-1997, SLICC-2012, and EULAR/ACR-2019 criteria [16,17,18,20]. The presence of IIF-ANA titer of 1:80 or more serves as an obligatory entry criterion of SLE by the EULAR/ACR-2019 criteria [20]. If the patient is positive for ANA, further testing for antigen-specific ANAs, such as dsDNA, Sjögren’s syndrome antigen A (SSA (Ro60)), Sjögren’s syndrome antigen B, Sm, and ribonuclear protein should be done. Although ANA is not unique to SLE, it is highly characteristic of SLE and can be used as a biomarker for screening, classification, diagnosis, prognosis, and staging [25,29]. ANA tests have high sensitivity, ranging from 90% to 95% in SLE patients [30] but a relatively low specificity as they can occur in 5–20% of healthy controls, especially in older people [31]. The sensitivity of ANA tests may be related to the early detection of SLE [32]. Although ANA tests are essentially universal for SLE, a negative ANA test cannot rule out SLE diagnosis. Choi et al. reported that 6.2% of patients with SLE were ANA-negative [33]. In fact, up to 30% of patients with SLE screened in clinical trials for new therapies are ANA-negative [34,35]. The inconsistency of ANA results in SLE may be due to variability in IIF-ANA assays, which have discrepant antigen properties (e.g., the combining capacity between molecularly cloned proteins and HEp-2 cells), the effects of nuclear antigens by HEp-2 cells, variability in laboratory routines, or different threshold in positive-ANA judgment [36,37,38]. Therefore, a standard ANA test with a consistent fine specificity, detection method, and laboratory protocol should be established in the future.

Although the IIF assay on HEp-2 cells is widely used for detecting ANA [25], enzyme-linked immunosorbent assay (ELISA) has also been used [29]. Antigens used in ELISA assays vary in source and composition, and they can be a mixture of proteins, DNA, and cell extracts [38]. IIF and ELISA are two kinds of techniques and have different sensitivities and specificities. IIF-ANA has a high sensitivity because multiple antibodies are detected simultaneously. However, this method also has some defects, such as being time-consuming, having a higher rate of false positives, and being difficult to categorize the specific antibody type. ELISA has a high sensitivity, as it uses purified antigens. Regardless of the test chosen, anyone testing for ANA should be familiar with the specific assay being used, including its specificity and sensitivity [29,39].

### 4.2. Serum Complement 3 (C3) and Complement 4 (C4)

Immune complexes can activate complements [40]. Serum C3 and C4 are widely used to assess the presence of biologically active immune complexes [41] and monitor disease activity. Low serum levels of C3 or C4 are considered immunological biomarkers in the SLICC-2012 SLE classification criteria. As one of the immunological criteria, low levels of both C3 and C4 are weighted higher than having low levels of either C3 or C4 alone in the EULAR/ACR-2019 classification criteria for SLE [22]. Patients with low levels of both C3 and C4 are more readily diagnosed with SLE than patients who exhibitlow C3 or C4, and patients with either low C3 or low C4 together with a positive ANA test showed 94.3% specificity for an SLE diagnosis, while patients with simultaneously low C3 and C4 levels combined with a positive ANA showed 97.6% specificity for an SLE diagnosis [42]. Furthermore, decreased levels of C3 and C4 can precede a clinically evident flare and positively correlate with SLE disease activity [43], especially in SLE complicated with renal or hematologic flares [44]. However, owing to the low specificity of C3 and C4 in diagnosing SLE, the reliability of C3 and C4 levels as biomarkers can be limited in diagnosing SLE and assessing disease activity in some patients, especially if used in isolation [24].

### 4.3. Anti-Nucleosome Antibodies (ANuA)

The prevalence of ANuA in SLE varies from 50% to 100%, and ANuA can be combined with clinical findings and other laboratory tests for diagnosing SLE and drug-induced lupus [45]. The presence of ANuA is related to glomerulonephritis and disease activity in SLE patients [45]. Moreover, the sensitivity of the ANuA assay for SLE is 61%, and the specificity is 94%. The overall positive likelihood ratio of ANuA is 13.81, and the negative likelihood ratio is 0.38. The probability that a subject with positive ANuA has SLE is 41 times greater than a subject with negative ANuA [46].

### 4.4. Erythrocyte Sedimentation Rate (ESR) and C-Reactive Protein (CRP)

In clinical practice, high ESR values, along with low CRP levels, are a key sign of inflammation in SLE and can be used for monitoring SLE disease activity [47,48]. ESR and CRP values are increased proportionally and simultaneously in a subgroup of SLE patients with serositis and/or arthritis [47]. ESR levels greater than25mm/hare strongly associated with SLE disease activity [49].

## 5. Organ-Specific SLE Biomarkers

SLE can cause multiple organ damage. Table 2 showed the biomarkers that could assess, monitor, and/or predict organ-specific involvement in patients with SLE, and this information would be helpful for organ-specific precision medicine for patients with SLE.

### 5.1. Biomarkers in Lupus Nephritis (LN)

Renal biopsy is the gold standard for diagnosing, classifying, and prognosing LN. However, it cannot be widely employed due to certain disadvantages, including it being an intrinsically invasive procedure, the risk of bleeding, and the possibility of sampling error [87]. Furthermore, a 10% to 20% misclassification risk may occur when conducting a fine needle percutaneous renal biopsy because of the possibility of not being able to penetrate the pathological location of renal or pathological error analysis [88]. In addition, serial biopsies cannot be conducted due to the invasive nature and potential complications associated with the procedure [89,90]. For these reasons, routine renal biopsy has been considered controversial and a question has been raised about whether it is absolutely required to diagnose LN [91].

#### 5.1.1. Serum Anti-dsDNA Antibodies

Anti-dsDNA antibodies are biomarkers that are associated with SLE disease activity [50,51], and they can predict the development of LN [51]. The level of anti-dsDNA antibodies can fluctuate over time [92] because of their association with SLE disease activity [38,93]. They can disappear during treatment and return during a flare, especially in active nephritis [94]. As one of the most characteristic ANA types, anti-dsDNA antibodies have a high specificity (96%) but low diagnostic sensitivity (52–70%) for SLE because of their transient appearance [18,52].

IIF using *Crithidialuciliae* as a substrate and ELISA are the most common assays for anti-dsDNA detection. The level of anti-dsDNA antibodies does not always correlate with active LN, and this makes anti-dsDNA an unreliable biomarker for assessing disease activity [92]. Although anti-dsDNA antibodies can exacerbate LN by being deposited in the kidney or driving cytokine production, the levels of anti-DNA antibodies do not necessarily correlate with active disease [92]. The reasons for anti-dsDNA antibody level variability may be the following: (1) antibody concentration varies with the SLE pathology over time, (2) antibodies may be deposited in renal tissue and not detectable in serum, (3) assays for anti-dsDNA antibodies are not sensitive and specific enough, (4) non-nephritic flares are not always accompanied by anti-dsDNA antibodies [95], and (5) not all subclasses of anti-dsDNA antibodies are pathogenic and associated with disease activity and renal disease [92,96].

#### 5.1.2. Serum Anti-SmAntibody

Presence of anti-Sm antibodies is included in SLE classification criteria and serves as a biomarker for SLE classification [18,20]. Anti-Sm antibodies are characteristic of SLE and have not been detected in other rheumatic diseases or in healthy individuals. Anti-Sm antibodies are correlated with SLE disease activity and are a highly specific diagnostic biomarker for SLE with a specificity of 99% but have a low sensitivity of 5–30% [1,18,26,53,54]. Moreover, anti-Sm antibodies are associated with LN, and their presence in patients with SLE indicates that LN may be acquired in the future [55,56]. In addition, high titers of anti-Sm antibodies were identified as predictor of silent LN [57] and may predict early poor outcomes in LN [56].

#### 5.1.3. Anti-C1q Antibodies

Hereditary C1q deficiency is strongly associated with SLE [97]. Anti-C1q antibodies result in decreased C1q, which may play a pathogenic role in the development of LN by inhibiting clearance of immune complexes and apoptotic bodies or by depositing immune complexes to the glomerular basement membrane [98,99]. Increased anti-C1q antibody titers predict renal flares in LN with an 81% to 97% sensitivity and a 71% to 95% specificity [58,59]. Moreover, anti-C1q titers are correlated with active LN, and the absence of anti-C1q is associated with a nearly 100% negative predictive value for the development of LN [60]. These reports suggest that anti-C1q antibodies may serve as a non-invasive biomarker for predicting renal flares. Unfortunately, anti-C1q antibodies have not been included in SLE classification criteria or for the clinical management of SLE because they lack a standardized laboratory assay [61]. In addition, anti-C1q antibodies can be found in other autoimmune diseases, including hypocomplementemic urticarial vasculitis (100%), Sjögren’s syndrome (26%), rheumatoid arthritis (19%), and Stevens-Johnson syndrome (14%), and even in apparently healthy individuals (3–5%) [61,98].

#### 5.1.4. Other Urinary Biomarkers

Urine is an easily and non-invasively obtainable biological sample and it directly reflects pathological changes in kidneys [68]. Hence, urinary biomarkers seem to be more promising than serum biomarkers. Proteinuria, protein/creatinine ratio in spot urine, and 24-h urine protein are conventional urinary biomarkers for LN. However, spot urine protein/creatinine ratio is not always a reliable estimate of 24-h proteinuria [62,63]. Therefore, new urinary biomarkers have been investigated. Various urine protein candidate biomarkers, including chemokines (monocyte chemoattractant protein-1, RANTES, interferon [IFN]-γ-inducible protein 10 (IP-10), C-X-C motif chemokine 16, and interleukin-8), cytokines (urinary tumor necrosis factor [TNF]-like weak inducer of apoptosis, interleukin-17, interleukin-6, adiponectin, transforming growth factor-beta, and osteoprotegerin [64]), growth factors, adhesion molecules (vascular cellular adhesion molecules 1 [65], and intracellular adhesion molecule1 [66]) have been evaluated as potential SLE biomarkers, but few of them have been independently validated. Moreover, urine angiopoetin-like 4, L-selectin, and TGF-β1 are biomarker candidates for tracking disease activity in LN [67]. A few points to note regarding urinary biomarkers include that their concentration can vary daily, their measurement could be affected by urinary infections—which should be ruled out, and they may not be specific for a particular disease since most inflammatory diseases share common molecular pathways [68].

### 5.2. Biomarkers for Skin Lesions in SLE

Skin lesions are typical clinical manifestations of SLE, and only a few biomarkers can be used for them. Our previous study reported that the ratio of aryl hydrocarbon receptor in Th17 cells to that in Treg (AhR ratio) is associated with SLE activity, and this ratio may be an independent risk factor for skin lesions in SLE [68]. Moreover, anti-SSA antibodies are associated with subacute cutaneous lupus [69]. The skin-targeted overexpression of vestigial-like family member 3 drives a proinflammatory gene expression program that leads to cutaneous lupus [70].

### 5.3. Biomarkers in Neuropsychiatric SLE (NPSLE)

NPSLE is one of the severe complications of lupus, which affects the central and peripheral nervous systems. Biomarkers for NPSLE are obtained from serum or cerebrospinal fluid (CSF) [71]. Antiphospholipid antibodies, including lupus anticoagulant, anticardiolipin, and anti-β2-glycoprotein I antibodies, are detected in serum and/or CSF are associated with NPSLE manifestations [71,72]. They are diagnostic biomarkers of NPSLE and used to make treatment decisions [71]. Moreover, the presence of antibodies against N-methyl-D-aspartate receptor (anti-NMDAR) in CSF are associated with central-nervous-system manifestations of NPSLE, such as an acute confusional state, anxiety disorders, cognitive dysfunctions, mood disorders, and psychosis, and it may distinguish patients with central NPSLE from peripheral NPSLE [76,77]. The level of serum anti-NMDAR is higher in patients with NPSLE than in SLE patients without NPSLE [77]. Antibodies against ribosomal proteins (anti-RibP) are a highly specific biomarker for diagnosing SLE and are associated with NPSLE [73]. The variability of anti-RibP test results from different assays and diagnostic platforms is the main challenge for its clinical use [73]. ELISA, immunodiffusion, and immunoblot are the main methods used to detect anti-RibP, and all of them have significant heterogeneity [73].

Increased levels of immunological biomarkers in CSF, such as interleukin-6, interleukin-8, interleukin-10, TNF-a, IFN-γ, monocyte chemoattractant protein-1, and IP-10 were associated with NPSLE [78,79]. Anti-U1 ribonucleoprotein antibodies were elevated in the CSF and sera of NPSLE patients [74], and they might cause NPSLE by inducing neurotoxic inflammatory mediators in the sheath [75].

### 5.4. Biomarkers for Cardiovascular Involvement in SLE

Cardiovascular disease (CVD) is one of the most important complications of SLE, and it is an important factor causing morbidity and mortality. The monocyte-to-high-density lipoprotein cholesterol ratio and low-density granulocytes-to-high-density lipoprotein cholesterol ratio are high in SLE patients with atheromatosis but not in CVD-free patients, and thus they are biomarkers for identifying CVD risk in SLE patients, even at the onset of disease [80]. Moreover, dysfunctional high-density lipoprotein is a key biomarker of accelerated atherosclerosis in lupus and may serve as a potential therapeutic biomarker for SLE patients with CVD [83]. High sensitivity of serum cardiac troponin T was the first identified biomarker that was independently associated with incident cardiovascular events in SLE patients [81,82]. Serum levels of antibodies against paraoxonase 1 and high-density lipoprotein are potential early biomarkers of endothelial damage and premature atherosclerosis in SLE, and thus they are useful therapeutic targets for preventing CVD in SLE patients [84]. In addition, serum levels of IgG-anticardiolipin antibodies [85] and E-selectin [86] are associated with CVD in lupus and correlated with disease activity.

## 6. Omics Approaches in SLE

Omics approaches provide opportunities for revealing new biomarkers for SLE to track the disease course with greater sensitivity and specificity. Omics, including the transcriptome (mRNA and single-cell), epigenome, genome, and metabolome, have been extensively used to gain insight into SLE [100,101,102].

### 6.1. Transcriptome in SLE

An integrative analysis of publicly available multi-omics data from the T cells, B cells, and peripheral blood cells of SLE patients demonstrated that 18 transcription factors were significantly enriched in all three types of cells [103]. The most prominent of them were related to type I IFN signaling, such as STAT1 and STAT2, which are two essential components of the IFN-stimulated gene factor 3 complex and can bind to the IFN-stimulated response element (ISRE) in the promoters of IFN-stimulated genes (ISGs) [103]. A single-cell transcriptomic analysis of renal stromal cells of LN patients demonstrated that the IFN and fibrosis characteristics of tubule cells might be associated with poorer response to therapy [104]. Using single-cell RNA sequencing, Nehar-Belaid et al. reported that an increased level of ISGs can distinguish cells from children with SLE from cells of healthy controls, and the high levels of ISGs are derived from transcriptionally defined subpopulations within major cell types, including monocytes, CD4^+^ and CD8^+^ T cells, natural killer cells, dendritic cells, B cells, and especially plasma cells. Subpopulations of enriched ISGs and/or monogenic lupus-associated genes can classify SLE activity [105]. Single-cell RNA sequencing was also used to analyze kidney samples from patients with LN, and it was found that local activation of B cells is correlated with an age-associated B-cell signature, and gene expression of immune cells in urine and kidneys was correlated, which indicates that urine might serve as a surrogate for kidney biopsies [106]. Although peripheral blood neutrophils may not be a robust biomarker, they were identified as a risk factor for LN by transcriptomic analyses [107]. Neutrophil-related transcriptome signatures associate with progression to active nephritis [108].

### 6.2. Epigenome in SLE

Epigenetic alterations play a key role in SLE pathogenesis through the dysregulation of gene expression. Epigenetic markers can be influenced by environmental factors. An elegant array of genome-wide approaches was employed to elucidate the epigenetic differences between B cell subsets in patients with SLE from similar subsets derived from healthy control subjects [109]. Epigenetic analyses of B cell subsets from patients with SLE demonstrate that the disease environment can shape the epigenetic networks of B cells [110]. These analyses also reveal the important connections between double-negative and ABC-like B cells [110]. The methylation level of the *IFI44L* promoter is a highly sensitive and specific diagnostic marker for SLE, and it can separate patients with SLE from healthy persons or patients with other autoimmune diseases [111].

### 6.3. Genome in SLE

Identifying genetic risk loci for SLE susceptibility has been a focus of study for a long time. Genome-wide association studies (GWAS) have been important for generating robust research in the genetics of lupus. Polygenic risk scores (PRS), calculated by GWAS data, showed a 30.3 times higher SLE risk in the patients of the highest decile compared to those in the lowest decile [112]. This suggests that PRS has a very strong impact on SLE susceptibility and may be used as a biomarker for SLE. Moreover, high genetic risk score (GRS) is reported to be associated with an increased risk of organ injury, renal dysfunction, and all-cause mortality in SLE disease [113]. All of these results indicate that assessing the genome may be useful for predicting the risk of getting SLE and its outcomes. Through expression quantitative trait locus (eQTL) analysis, analysis of the association between genetic variants and gene expression levels, we have recently reported that polygenic SLE risks fit closely with the eQTLs of naïve and unswitched memory B cells [114]. In addition, integrin alpha M (*ITGAM*) was confirmed as a susceptibility gene of LN [115], and the low-frequency risk alleles of the *ITGAM* gene were identified as risk factors that relate to disease susceptibility and severe manifestations of SLE [116].

### 6.4. Metabolome in SLE

The metabolome is now considered to play a key role in SLE due to developments in mass-spectrometry-based metabolic flux analysis. The fecal metabolomes of 29 SLE patients and 30 healthy controls were analyzed by gas chromatography-mass spectrometry [117]. The results suggest that L-valine, pyrimidine, erucamide, and L-leucine are excellent potential diagnostic biomarkers for SLE, and alterations of fecal metabolites are closely correlated with SLE [117]. Proteomic profiling of kidney tissues from SLE revealed that coronin-1A is a biomarker of LN, and the level of it in serum can distinguish LN patients from SLE patients without nephritis with 100% sensitivity and 100% specificity [118]. Moreover, granzyme B is increased in the serum and kidneys of patients with SLE, and it is correlated with a poor prognosis of LN [119].

## 7. Challenges in Biomarker Discovery

Biological variability is unavoidable, and it is a great challenge that has been faced in clinical, and especially in ‘omics’, studies. The variability of assessment is a key factor affecting biomarker studies. For instance, many biomarkers for SLE have not yet demonstrated sufficient sensitivity, specificity, or predictive power for clinical use, and low measurement accuracy and reproducibility also limit their usability. Moreover, the lack of validation cohorts also limits the estimation of any biomarkers that are identified. To improve biomarker studies and measurement accuracy and reproducibility, research should be carefully designed: sample size should be calculated based on sample type and technique employed, parameters should be specific, and appropriate statistical analysis should be conducted [120].

## 8. Conclusions and Future Perspectives

More robust immunological biomarkers are needed to better understand disease progression in individuals with SLE, including non-organ-specific SLE biomarkers and organ-specific SLE biomarkers. Since no single biomarker can be sensitive and specific enough for SLE, multiple biomarkers combined through mathematical models may be a good idea for assessing SLE. Moreover, advanced computational methods are required to analyze large datasets and discover novel biomarkers.

## Figures and Tables

**Table 1:** Biomarkers for SLE in the defined criteria of ACR-1997, SLICC-2012, and EULAR/ACR-2019.

Biomarkers	ACR-1997 Criteria	SLICC-2012 Criteria	EULAR/ACR-2019 Criteria
Proteinuria	Persistent proteinuria > 0.5 g/24 h or >3+, if quantitation not performed	Urine protein to creatinine ratio (or 24-h urine protein) representing 500 mg protein/24 h	Proteinuria > 0.5 g/24 h by 24-h urine or equivalent spot urine protein to creatinine ratio
Urinary casts	Cellular casts may be red cell, hemoglobin, granular, tubular, or mixed	Red blood cell casts	—
Hemolytic anemia	Hemolytic anemia with reticulocytosis	Direct Coombs’ test in the absence of hemolytic anemia	Evidence of hemolysis, such as reticulocytosis, low haptoglobin, elevated indirect bilirubin, elevated LDH, and positive Coombs’ (direct antiglobulin) test
White blood cell count	White blood cell count < 4000/mm^3^ on 2 or more occasions; OR Lymphocyte count < 1500/mm^3^ on 2 or more occasions	White blood cell count < 4000/mm^3^ at least once, in the absence of other known causes such as Felty’s syndrome, drugs, and portal hypertension; OR Lymphocyte count < 1000/mm^3^ at least once, in the absence of other known causes such as corticosteroids, drugs, and infection	White blood cell count < 4000/mm^3^
Platelet count	Platelet count < 100,000/mm^3^ in the absence of offending drugs	Platelet count < 100,000/mm^3^ at least once, in the absence of other known causes such as drugs, portal hypertension, and thrombotic thrombocytopenic purpura immunologic criteria	Platelet count < 100,000/mm^3^
Sm antibody	Presence of antibodies to Sm nuclear antigen	Presence of antibodies to Sm nuclear antigen	anti-Sm antibodies
Serologic text for syphilis	False positive serologic test for syphilis known to be positive for at least 6 months and confirmed by *Treponernapallidun* immobilization or fluorescent treponemal antibody absorption test	—	—
Antinuclear antibody levels	An abnormal titer of antinuclear antibody by immunofluorescence or an equivalent assay at any point in time and in the absence of drugs known to be associated with “drug-induced lupus” syndrome	ANA level above laboratory reference range	ANA at a titer of ≥1:80 on HEp-2 cells or an equivalent positive test at least once; testing by immunofluorescence on HEp-2 cells or a solid-phase ANA screening immunoassay with at least equivalent performance is highly recommended
DNA antibody	Antibody to native DNA in abnormal titer	Anti-dsDNA antibody level above laboratory reference range (or 2-fold the reference range if tested by ELISA)	Anti-dsDNA antibodies in an immunoassay with demonstrated ≥ 90% specificity for SLE against relevant disease controls
CH50	CH50	Low CH50	—
Complement 3	Complement 3	Low complement 3	Low complement 3
Complement 4	Complement 4	Low complement 4	Low complement 4
Complement 2	Complement 2	—	—
Antiphospholipid antibody	Antiphospholipid antibody positivity	Antiphospholipid antibody positivity as determined by any of the following: positive test result for lupus anticoagulant; false-positive test result for rapid plasma regain; medium- or high-titer anticardiolipin antibody level (IgA, IgG, or IgM); positive test result for anti-2-glycoprotein I (IgA, IgG, or IgM)	Anticardiolipin antibodies (IgA, IgG, or IgM) at medium or high titer (>40 APL, GPL, or MPL, or >the 99th percentile) or positive anti-β2GPI antibodies (IgA, IgG, or IgM) or positive lupus anticoagulant

ACR: American College of Rheumatology; ANA: antinuclear antibody; anti-β2GPI: anti-β2-glycoprotein I; CH50: total complement activity; dsDNA: double-stranded DNA; ELISA: enzyme-linked immunosorbent assay; EULAR: the European Alliance of Associations for Rheumatology; IgA: immunoglobulin A; IgG: immunoglobulin G; IgM: immunoglobulin M; LDH: lactate dehydrogenase; SLE: systemic lupus erythematosus; SLICC: Systemic Lupus International Collaborating Clinics; Sm: Smith.

**Table 2:** Clinical and immunological biomarkers of organ-specific damage in SLE.

Organ-Specific Damage in SLE	Biomarkers	Sample Type	Key Points	Refs
Lupus nephritis	Anti-dsDNA antibodies	Serum	Associated with SLE disease activity and can predict the development of LN; high specificity (96%), low diagnostic sensitivity (52–70%).	[18,50,51,52]
	Anti-Sm antibodies	Serum	Correlates with SLE disease activity and LN; highly specific diagnostic biomarker for SLE with a specificity of 99% but with a low sensitivity of 5–30%; high titers of anti-Sm antibodies predict silent LN; predict early poor outcomes in LN.	[1,18,26,53,54,55,56,57]
	Anti-C1q antibodies	Serum	Increased anti-C1q antibody titers predict renal flares in LN with an 81–97% sensitivity and a 71% to 95% specificity; anti-C1q titerscorrelates with active LN, and the absence of anti-C1q is associated with a nearly 100% negative predictive value for the development of LN; Standardized laboratory assay has not been established.	[58,59,60,61]
	Proteinuria; Protein/creatinine ratio; 24-h urine protein	Urine	Conventional urinary biomarkers for LN. Spot urine protein/creatinine ratio is not always reliable estimate of 24-h proteinuria.	[62,63]
	Chemokines (MCP-1, IL-8, RANTES, IP-10, CXCL-16); Cytokines (TGF-β, IL-17,uTWEAK, adiponectin, IL-6, osteoprotegerin); Adhesionmolecules (VCAM-1, ICAM-1)	Urine	Evaluated as potential SLE biomarkers, but few of them have been independently validated.	[64,65,66]
	Angiopoetin-like 4; L-selectin; TGF-β1	Urine	Biomarker candidates for tracking disease activity in LN.	[67]
Skin lesions	AhR ratio	Serum	Associated with SLE activity and may be an independent risk factor for skin lesions in SLE.	[68]
	Anti-SSA antibodies	Serum	Associated with subacute cutaneous lupus.	[69]
	VGLL-3	Serum	Leads to cutaneous lupus.	[70]
NPSLE	Lupus anticoagulant antibodies; Anticardiolipin antibodies; Anti-β2-glycoprotein I antibodies	Serum or CSF	Associated with NPSLE manifestations; diagnostic biomarkers of NPSLE and used to make treatment decisions.	[71,72]
	Anti-RibP	Serum or CSF	Highly specific biomarker in the diagnosis of SLE and associated with NPSLE.	[73]
	Anti-U1 ribonucleoprotein antibodies	Serum or CSF	Elevated in CSF and sera of NPSLE patients; might cause NPSLE.	[74,75]
	Anti-NMDAR	CSF	Associated with central nervous system manifestations of NPSLE.	[76,77]
	IL-6, 8, 10, TNF-a; IFN-γ; MCP-1; IP-10	CSF	Associated with NPSLE.	[78,79]
CVD	LHR; MHR	Serum	Predicts CVD risk in SLE patients, even at the onset of disease.	[80]
	Cardiac troponin T	Serum	Independently associated with incident cardiovascular events in SLE patients.	[81,82]
	Paraoxonase 1 and HDL	Serum	Key biomarker of accelerated atherosclerosis in lupus and may serve as a potential therapeutic biomarker for SLE patients with CVD; early biomarkers of endothelial damage and premature atherosclerosis in SLE; therapeutic targets for preventing CVD in SLE patients.	[83,84]
	IgG-anticardiolipin antibodies; E-selectin	Serum	Associated with CVD in SLE and correlated with disease activity.	[85,86]

AhR ratio: the ratio of aryl hydrocarbon receptor in Th17 cells to that in Treg; Anti-NMDAR: N-methyl-D-aspartate receptor; CSF: cerebrospinal fluid; CVD: cardiovascular disease; CXCL-16: C-X-C motif chemokine 16; HDL: high-density lipoprotein; ICAM-1: intracellular adhesion molecule1; IL: interleukin; IP-10: interferon[IFN]-γ-inducible protein 10; LHR: low-density granulocytes-to-high-density lipoprotein cholesterol ratio; MCP-1: monocyte chemoattractant protein-1; MHR: monocyte-to-high-density lipoprotein cholesterol ratio; NPSLE: neuropsychiatric systemic lupus erythematosus; RANTES: regulated upon activation, normal T-cell expressed and secreted; TGF-β: transforming growth factor-beta; uTWEAK: urinary tumor necrosis factor [TNF]-like weak inducer of apoptosis; VCAM-1: vascular cellular adhesion molecules 1; VGLL-3: vestigial-like family member 3.
